# Untargeted metabolomics and transcriptomics identified glutathione metabolism disturbance and PCS and TMAO as potential biomarkers for ER stress in lung

**DOI:** 10.1038/s41598-021-92779-8

**Published:** 2021-07-19

**Authors:** Zijing Wang, Peng Ma, Yisa Wang, Biyu Hou, Can Zhou, He Tian, Bowen Li, Guanghou Shui, Xiuying Yang, Guifen Qiang, Chengqian Yin, Guanhua Du

**Affiliations:** 1 State Key Laboratory of Bioactive Substance and Function of Natural Medicines, Institute of Materia Medica, Chinese Academy of Medical Sciences and Peking Union Medical College and Beijing Key Laboratory of Drug Target and Screening Research, Beijing, 100050 China; 2 College of Pharmacy, Harbin University of Commerce, Harbin, 510006 China; 3 Beijing Anzhen Hospital, Capital Medical University, Beijing, 100029 China; 4 State Key Laboratory of Molecular Developmental Biology, Institute of Genetics and Developmental Biology, Chinese Academy of Sciences, Beijing, 100101 China; 5 LipidALL Technologies Ltd., Changzhou, China

**Keywords:** Biochemistry, Computational biology and bioinformatics

## Abstract

Endoplasmic reticulum (ER) stress is a cellular state that results from the overload of unfolded/misfolded protein in the ER that, if not resolved properly, can lead to cell death. Both acute lung infections and chronic lung diseases have been found related to ER stress. Yet no study has been presented integrating metabolomic and transcriptomic data from total lung in interpreting the pathogenic state of ER stress. Total mouse lungs were used to perform LC–MS and RNA sequencing in relevance to ER stress. Untargeted metabolomics revealed 16 metabolites of aberrant levels with statistical significance while transcriptomics revealed 1593 genes abnormally expressed. Enrichment results demonstrated the injury ER stress inflicted upon lung through the alteration of multiple critical pathways involving energy expenditure, signal transduction, and redox homeostasis. Ultimately, we have presented p-cresol sulfate (PCS) and trimethylamine N-oxide (TMAO) as two potential ER stress biomarkers. Glutathione metabolism stood out in both omics as a notably altered pathway that believed to take important roles in maintaining the redox homeostasis in the cells critical for the development and relief of ER stress, in consistence with the existing reports.

## Introduction

Endoplasmic reticulum (ER) plays an important role in protein processing and is abundant in cells with active secretive functions. As the unfolded and misfolded protein build up in the lumen of ER, the cell can initiate unfolded protein response (UPR) that work to rectify the stress by increasing the protein folding capacity and inhibiting overall protein synthesis. If the stress is not relieved, however, the cell can then be guided onto apoptotic pathways that eventually end in cell death. COVID-19 virus has been shown to cause^1^ or elevate^2^ ER stress in the course of disease, and a trusted therapy of respiratory infections, Lian Hua Qing Wen, has been proved to attenuate lung injury through inhibiting ER stress^3^. Apart from acute lung infections, chronic lung diseases such as asthma, pulmonary fibrosis and chronic obstructive pulmonary disease (COPD) had ER stress as a common feature^4^, and lung cancer, which is the leading death-causing cancer in both China^5^ and the United States^6^, has also been found to be related with ER stress through the changes of redox environment in the lumen^4^. A traditional Chinese medicine for cancer treatment, Rhizoma Arisaematis, has been shown to exert its cytotoxicity effect in lung cancer A549 cell line by presumably the induction of ER stress by agglutinin isolated from the herb that led cells onto apoptosis and autophagy pathways^7^. The fundamental roles ER plays as well as the ubiquitous identification of ER stress in lung pathologies made ER stress a cellular target state worthy of study in lung diseases.

Metabolomics and transcriptomics are two methods of profiling that have been frequently used to provide information regarding pathological phenotypes^8^ or for the aim of pathogenesis studies^9^, drug-target identification^10^, and biomarker recognition^11^ etc. Liquid chromatography–mass spectrometry (LC–MS), as a robust and informative tool in identifying and quantifying molecules in mixtures, could be used to get reliable data regarding the metabolites in tissues, making metabolic comparison between phenotypes possible. RNA sequencing (RNA-seq) on the other hand, provides the relative expression levels of genes that imply their roles in the target alterations (i.e., ER stress in this particular study). Cristea et al.^12^ have shown by transcriptomic profiling that MEK5/ERK5 kinase axis controls lipid metabolism in small-cell lung cancer. Nojima et al.^13^ revealed potential diagnostic biomarker candidates for lung fibrosis by metabolomic analysis of fibrotic mice in combination with public RNA-Seq human lung data. Through means of multi-omics (including RNA-seq), Suzuki et al.^14^ identified a stress response module as a promising drug intervention target in lung cancer cells, and stressed the potential of multi-omics in developing anticancer drugs rationally and efficiently.

In the current study, we analyzed the effects of ER stress in the lung utilizing both transcriptomic and metabolomic methods. After the induction of ER stress in C57BL/6J mice by intraperitoneal injection of tunicamycin, we performed untargeted metabolomic analysis (by LC–MS) in lung tissues to identify the differentially-regulated metabolites as well as reveal the enriched pathways in follow-up data interpretation. Transcriptomic analysis based on RNA-seq results was carried out in the same tissues to unravel the changes in gene expression and biological processes in response to ER stress. Results of both omics were then taken together in hope of finding molecules, pathways or biological processes playing central roles in ER stress. Collectively, we comprehensively evaluated the genetic and metabolic alteration in ER stressed lung samples aiming to shed light on the complex pathological mechanism as well as identify potential biomarkers of ER stress, which has been proven relative to multiple lung injury and diseases.

## Results

### ER stress was successfully induced with lung injury

**Figure 1:**
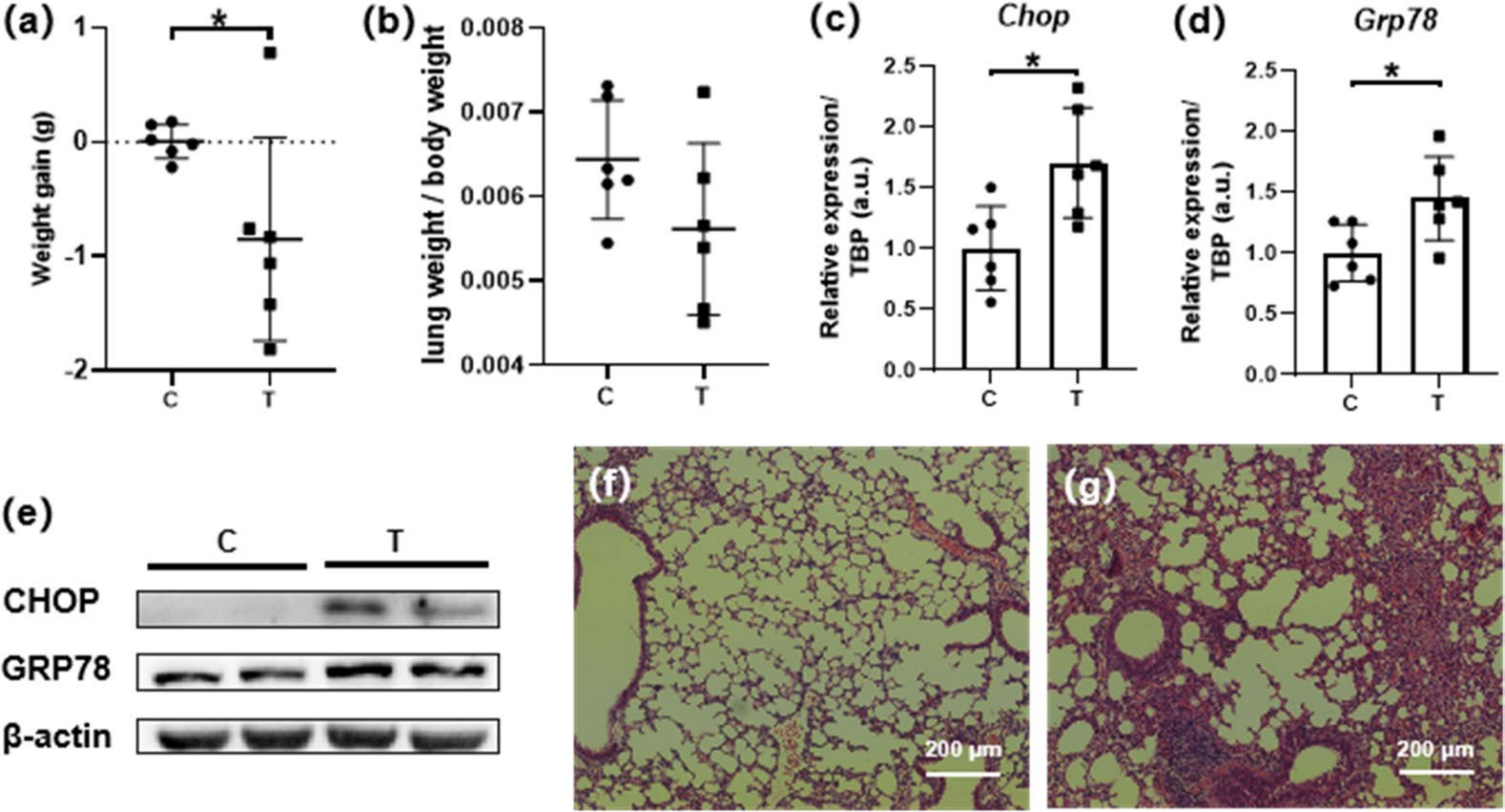
Successful induction of ER stress in lung of C57BL/6J mice. Abnormal weight loss (**a**) of T group was identified compared to C group, with no significant change in lung coefficient (**b**), elevated expression of marker proteins CHOP and GRP78 in T group was proven by qPCR (**c**,**d**) as well as western blot (**e**), pathological damages in T group at tissue level were revealed by HE staining of lung histologic sections [(**f**) C, (**g**) T]. All data were presented as mean ± SEM, n = 6. *P < 0.05. *C* Control, *T* Tunicamycin.

Tunicamycin induces UPR by inhibiting protein glycosylation^15^ and thus has been widely used as an ER stressor^16–18^. To observe the effect of ER stress in lung, we injected tunicamycin intraperitoneally in mice. As a result, the average body weight of the mice in model group (T) decreased 0.85 g (3.6%, *P* = 0.043) during the 12 h of ER stress induction by tunicamycin while no significant change in body weight was found in the control group (Fig. 1a), and the organ coefficient for lung was of no significant difference (Fig. 1b). More importantly, the presence of ER stress was indicated by elevation of ER stress-related marker genes and proteins (CHOP^18,19^ and GRP78^20,21^) detected in lung samples by qPCR as well as western blot (Fig. 1c–e). Besides, pathological changes were evident in the lung sections stained by HE. Focal destruction of lung tissue structure, alveolar epithelial hyperplasia and focal inflammatory cells infiltration could be seen near the bronchus in T group sections, while no pathological changes were identified in C group samples (Fig. 1f,g). Taken together, ER stress was successfully induced by tunicamycin with lung injury.

### Untargeted metabolomics revealed aberrantly-regulated metabolites and pathways

**Figure 2:**
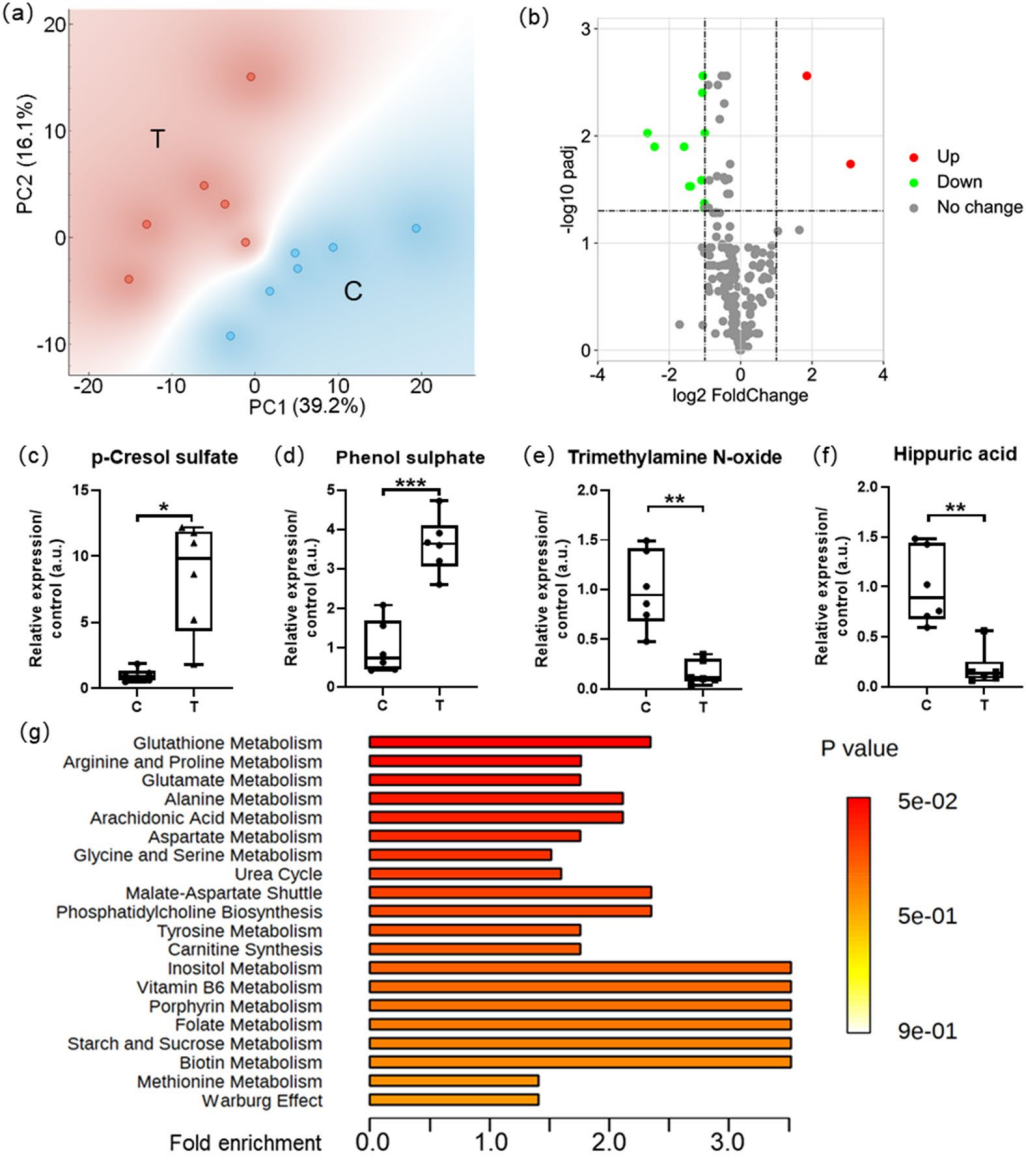
Untargeted metabolomics revealed aberrantly-regulated metabolites and pathways. PCA results (**a**) suggested good separation between data groups, volcano plot (**b**) presented aberrantly-regulated metabolites with padj < 0.05, fold change (T/C) > 2 or fold change reciprocal (reciprocal of fold change value) > 2, boxplots demonstrated the top 4 metabolites: PCS (**c**), phenol sulphate (**d**), TMAO (**e**), hippuric acid (**f**) with the most significant differences (fold change) between groups, top 20 enriched pathways (**g**) presented according to results of MSEA. All data were presented as mean ± SEM, n = 6. *P < 0.05; **P < 0.01; ***P < 0.001. *C* Control, *T* Tunicamycin.

Overall, 210 metabolites were identified in the 6 lung samples of each group. The results of principal component analysis (PCA) suggested good separation between groups (Fig. 2a). There were collectively 16 metabolites deemed abnormal according to our restrictions with the hypothesis test results (Fig. 2b), and were orderly listed in Table 1 according to their fold change values. P-cresol sulfate (PCS), phenol sulphate and 3-oxododecanoic acid were the abnormally up-regulated metabolites with average fold change of 8.4, 3.6 and 2.1 while the trimethylamine N-oxide (TMAO) as well as 12 other metabolites were detected of lower levels in the T group tissue than normally expected (refer to the values in C group) (Fig. 2c–f, Table 1).

**Table 1:** 16 metabolites deemed abnormal in metabolomics.

Metabolite	*P*-value	padj	Fold change	Fold reciprocal
p-Cresol sulfate	0.0068	0.0122	8.45	0.12
Phenol sulphate	0.0001	0.0011	3.62	0.28
3-Oxododecanoic acid	0.0235	0.0269	2.06	0.49
*N*-Acetylvaline	0.0005	0.0022	0.50	2.01
Alpha-Linolenic acid	0.0467	0.0467	0.49	2.02
d-Phenyllactic acid	0.0106	0.0141	0.49	2.02
sn2 LysoPC(18:1)	0.0117	0.0144	0.49	2.02
l-Aspartic acid	0.0003	0.0016	0.48	2.08
4-Pyridoxic acid	0.0002	0.0015	0.48	2.10
LysoPC(20:4)	0.0385	0.0410	0.47	2.11
5-methoxy-L-tryptophan	0.0068	0.0122	0.47	2.14
LysoPC(18:2)	0.0087	0.0126	0.38	2.64
sn2 LysoPC(18:2)	0.0079	0.0126	0.37	2.71
LysoPE(16:1)	0.0031	0.0072	0.33	3.00
Hippuric acid	0.0020	0.0063	0.19	5.31
Trimethylamine N-oxide	0.0024	0.0063	0.16	6.10

Restrictions: padj < 0.05, fold change (T/C) > 2 or fold change reciprocal (reciprocal of fold change value) > 2.

*C* Control, *T* Tunicamycin.

In order to further understand the in vivo metabolic alteration after 12 h of tunicamycin treatment, metabolite set enrichment analysis (MSEA) was carried out in reference to the database of pathway-associated metabolite sets (SMPDB). Out of the 51 metabolite sets with hits (metabolites with changes of statistical significance) no less than 2, glutathione metabolism was found to be the most significantly altered pathway (raw *P* = 0.049) relative to ER stress. There were 4 hit compounds (glycine, l-glutamic acid, l-alanine and oxidized glutathione) up-regulated in the pathway of glutathione metabolism and the fold enrichment for the whole pathway was 2.38. The top 20 metabolic pathways (Fig. 2g) were presented according to MSEA results, with detailed information shown in Supplementary Table S3.

### RNA sequencing revealed multiple genes abnormally regulated

**Figure 3:**
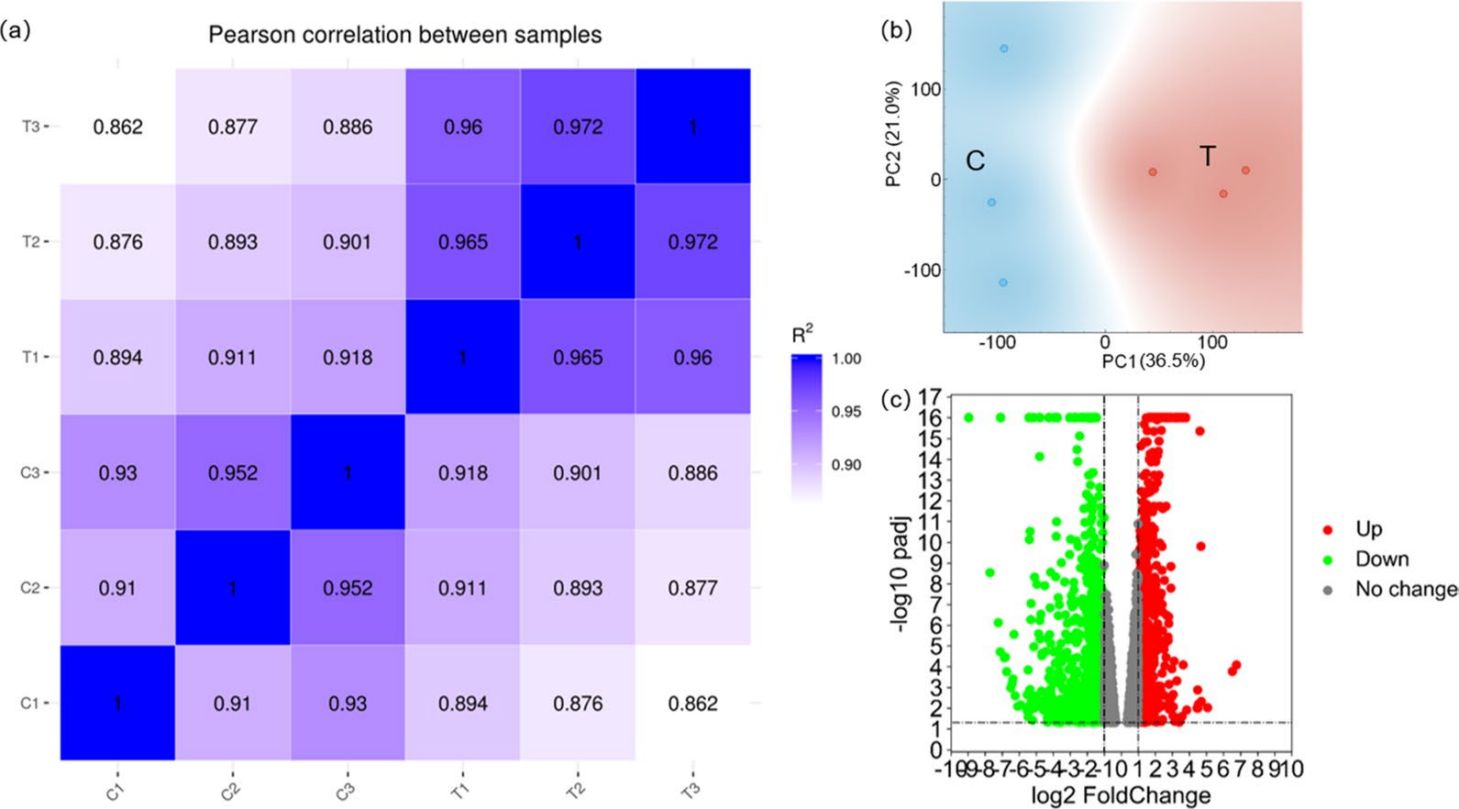
RNA-seq revealed 1593 genes abnormally regulated. Pearson correlation analysis (**a**) and PCA results (**b**) suggested good separation between the two data groups, volcano plot (**c**) signified aberrantly-regulated genes with padj < 0.05, fold change (T/C) > 2 or fold change reciprocal (reciprocal of fold change value) > 2. *C* Control, *T* Tunicamycin.

Pearson correlation analysis (Fig. 3a) between samples as well as PCA plot (Fig. 3b) demonstrated good separation between the two data groups. 1593 genes were found up-regulated or down-regulated in the T group compared to C group as shown in the volcano plot with the colored dots (Fig. 3c). Top 20 aberrantly expressed genes were orderly listed in Table 2 based on their padj values.

**Table 2:** Top 20 genes up-regulated or down-regulated in the RNA-seq results.

Gene name	*P*-value	padj	Fold change	Fold reciprocal
Galnt15	5.2E−06	6.2E−05	6.86	0.15
Adam3	6.2E−06	6.2E−05	2.07	0.48
Fam181a	1.5E−05	1.0E−04	0.14	7.00
S100g	2.1E−05	1.1E−04	0.27	3.74
Cth	3.7E−05	1.5E−04	2.45	0.41
Dnase1l3	5.7E−05	1.6E−04	0.47	2.11
Kcnn4	6.0E−05	1.6E−04	0.34	2.98
Dmkn	6.4E−05	1.6E−04	0.29	3.45
Gm35394	7.0E−05	1.6E−04	12.78	0.08
Vipr2	7.9E−05	1.6E−04	3.97	0.25
Slc6a9	9.2E−05	1.6E−04	4.11	0.24
Sesn2	1.0E−04	1.6E−04	2.04	0.49
Pebp1	1.1E−04	1.6E−04	0.50	2.00
Tbx4	1.1E−04	1.6E−04	2.07	0.48
Rpl31-ps13	1.4E−04	1.6E−04	0.35	2.88
Fam229b	1.5E−04	1.6E−04	0.41	2.44
Ak1	1.5E−04	1.6E−04	0.33	3.06
Igkv13-85	1.6E−04	1.6E−04	0.04	25.11
Serpina9	1.6E−04	1.6E−04	0.13	7.95
Rpl27-ps3	1.6E−04	1.6E−04	0.47	2.14

Restrictions: padj < 0.05, fold change (T/C) > 2 or fold change reciprocal (reciprocal of fold change value) > 2, records of 0 average FPKM in either group were removed, top 20 selected based on *P*-values.

*C* Control, *T* Tunicamycin.

### Pathways of interest found by enrichment analysis of RNA-seq results

**Figure 4:**
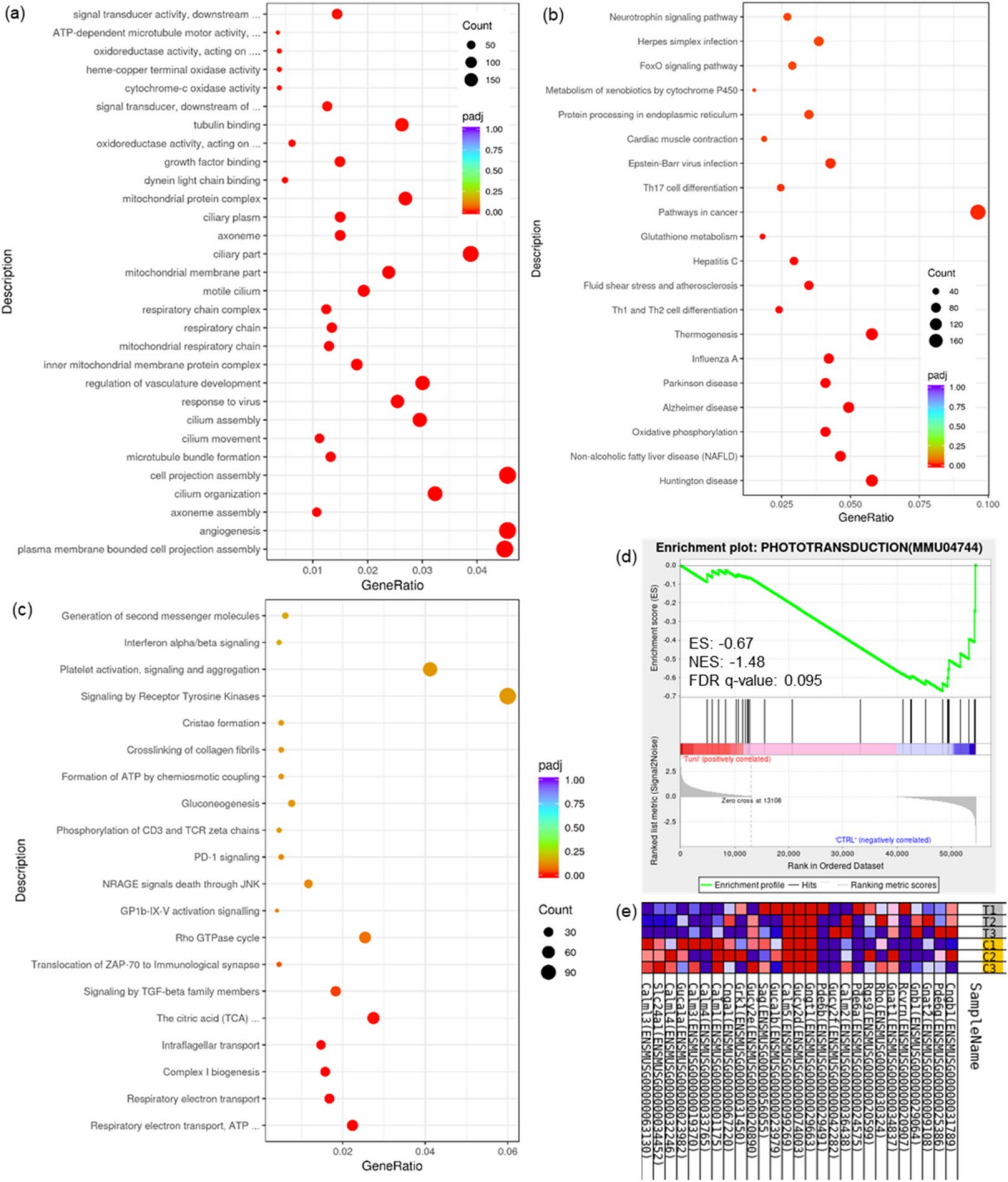
Multiple pathways aberrantly-regulated revealed by enrichment analyses. Functional enrichment results based on GO (**a**), KEGG (**b**) and Reactome (**c**) database were presented as dot bubble graphs (pathways with ascending significance from top to bottom), GSEA results revealed phototransduction pathway as the most prominently enriched process, up-regulated in response to ER stress: enrichment plot (**d**) profiling the running enrichment score (ES) and positions of gene set members on the rank ordered list, along with the heatmap (**e**) showing expression levels of the probed genes involved in the pathway of phototransduction. C/CTRL: Control, T/TUNI: Tunicamycin. The concealed part of the pathways’ names in (**a**): signal transducer activity, downstream of receptor; ATP-dependent microtubule motor activity, minus-end-directed; oxidoreductase activity, acting on a heme group of donors, oxygen as acceptor; signal transducer, downstream of receptor, with serine/threonine kinase activity; oxidoreductase activity, acting on peroxide as acceptor (from top to bottom); in (**c**): TCA cycle and respiratory electron transport; respiratory electron transport, ATP synthesis by chemiosmotic coupling, and heat production by uncoupling proteins.

Functional enrichment based on Gene Ontology (GO) database was carried out and pathways of significant variation in three sub-divisions of biological process (BP), cellular component (CC), molecular function (MF) were identified (Fig. 4a). 533 pathways (412 in BP, 80 in CC, 41 in MF) were considered to be statistically significant (padj < 0.05). Basic functions such as cell replication and movement (angiogenesis, cilium organization, growth factor binding, etc.), as well as energy expenditure (pathways such as respiratory chain that involve mitochondria) were all subject to the influence of tunicamycin-induced ER stress. 23 pathways were deemed significant in Kyoto Encyclopedia of Genes and Genomes^22–24^ (KEGG)-based enrichment (Fig. 4b) that included Huntington disease, non-alcoholic fatty liver disease (NAFLD), influenza A, and among others, glutathione metabolism in consistence with the results of untargeted metabolomics, signifying the disturbance of cellular redox homeostasis in state of ER stress. While in the functional enrichment based on Reactome database (Fig. 4c), 7 pathways came out as the significantly influenced, including cellular respiratory pathways like electron transport, tricarboxylic acid (TCA) cycle, complex I biogenesis, as well as intraflagellar transport and cancer-related pathways (regarding transforming growth factor (TGF)-beta, zeta-chain-associated protein (ZAP)-70).

GSEA was utilized in reference to the three previously mentioned gene set databases in order to reveal pathways of interest without the input limitation of alteration significance. GSEA based on GO, KEGG and Reactome enrichment gave respectively 485 and 208, 19 and 19, 74 and 32 significantly enriched (at nominal *P*-value < 1%) pathways in the two phenotypes (tunicamycin and control) and ultimately one gene set with false discovery rate (FDR) value less than 25% in the phenotype of control from KEGG-based enrichment: phototransduction (Fig. 4d,e). Yet this pathway was not found of notable significance in the enrichment results of metabolomics above.

### Informative PPI networks built based on RNA-seq results

**Figure 5:**
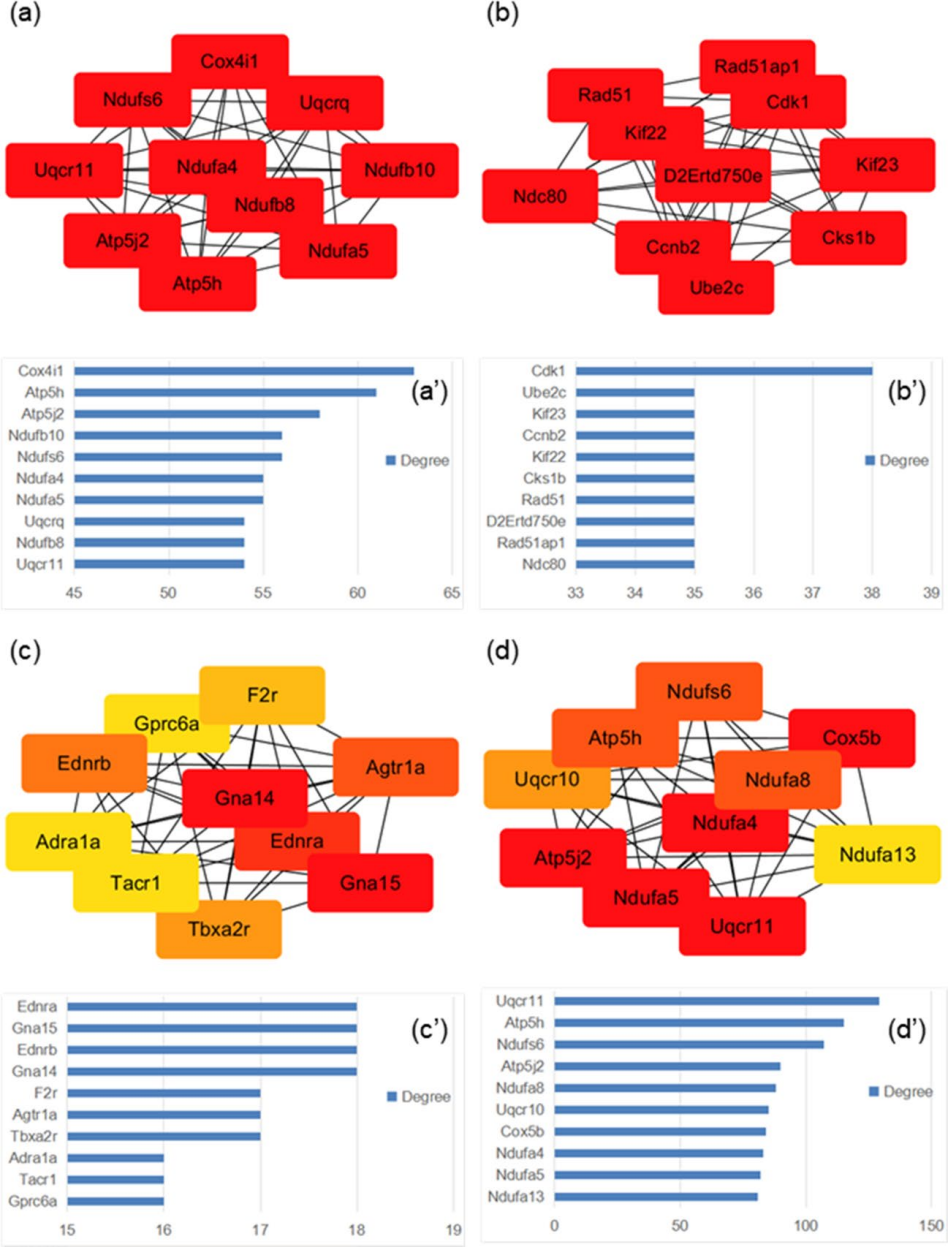
Protein–protein interactions were integrated in the Cytoscape software in the form of PPI networks. Central nodes (proteins with top 10 MCC values calculated by cytoHubba in each of the four clusters: (**a**–**d**) with degree values shown in (a′–d′), four clusters from which the central nodes were selected included top 3 PPI network clusters presented by MCODE analysis as well as the whole PPI network.

Protein–protein interactions were integrated in the Cytoscape software in the form of PPI networks. According to MCODE scores calculated by the plugin, MCODE, multiple clusters (Supplementary Figs. S1–3) were selected from the whole PPI network. The top 10 nodes (Fig. 5a–d) from each of the three clusters selected as well as the whole network were presented with their degree values (Fig. 5a′–d′). The clustered nodes (proteins/genes) signified their close relationship, whether through direct interactions in forming of a bigger protein complex, or through the involvement in a consistent pathway or biological process. These nodes signified cellular respiration greatly altered in the state of ER stress, demonstrating its deleterious effect on cells.

### Combination of the two omics revealed pathways influenced by ER stress

**Figure 6:**
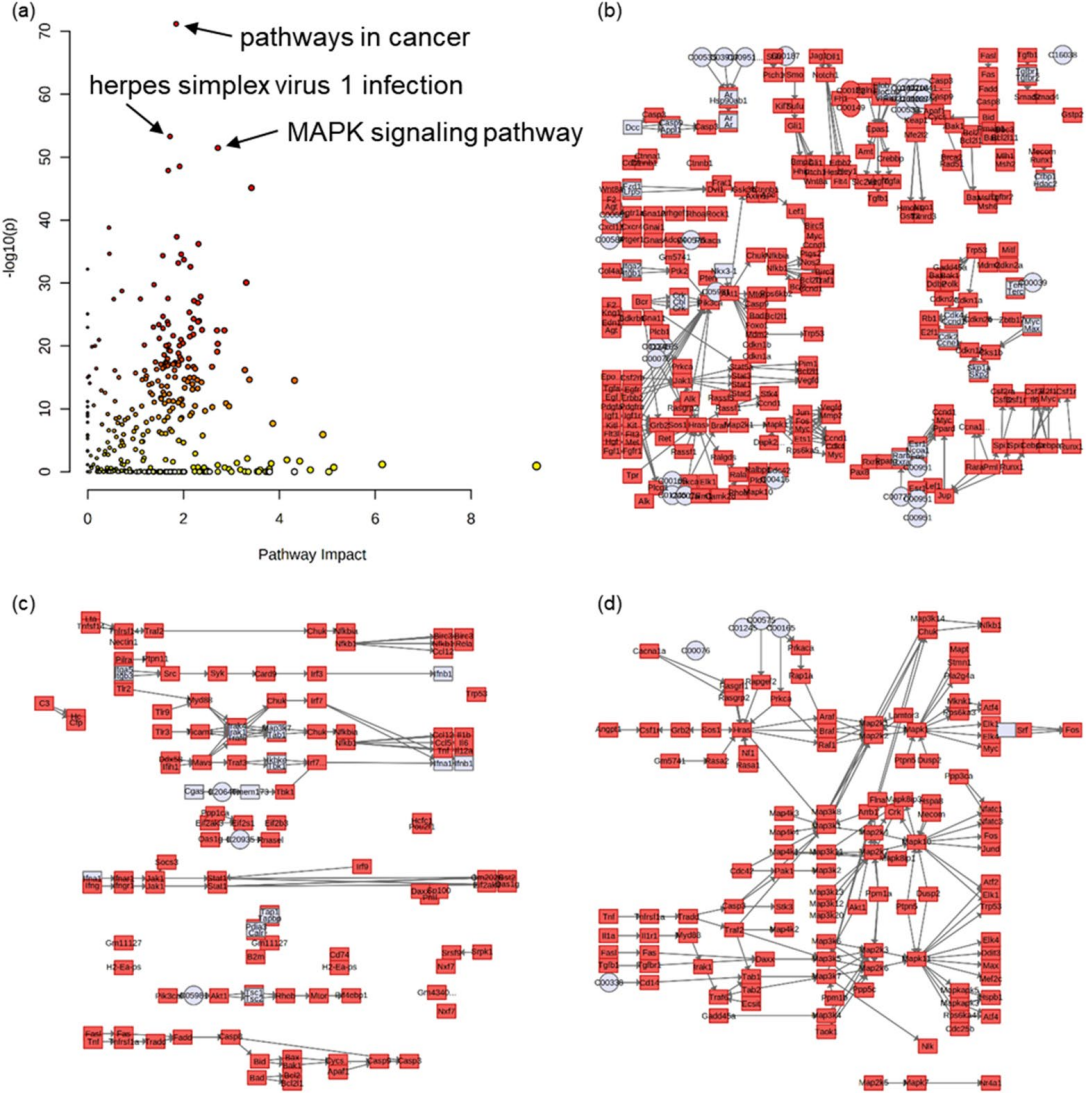
Integrative analysis of metabolomics and transcriptomics revealed pathways significantly varied. Overview (**a**) of the pathways was presented, and the most significantly altered pathways (pathways with lowest *P*-values) were pathways in cancer (**b**, − log10(p) = 71.2, impact = 1.85), herpes simplex virus 1 infection (**c**, − log10(p) = 53.3, impact = 1.72) and MAPK signaling pathway (**d**, − log10(p) = 51.5, impact = 2.72).

Significantly varied metabolites and genes were submitted in running joint-pathway analysis in Metaboanalyst. Out of the 324 biological processes identified (Fig. 6a), 221 and 229 pathways had *P*-value < 0.05 and FDR < 25% respectively. The top three pathways based on the calculation of *P*-value were pathways in cancer (Fig. 6b), herpes simplex virus 1 infection (Fig. 6c) and mitogen-activated protein kinase (MAPK) signaling pathway (Fig. 6d). The pathway of glutathione metabolism, found prominent in both omics, was also significantly enriched (*P* = 0.04, FDR = 0.06) in the integrative analysis and the impact value was 3.12. On the other hand, however, the variation of phototransduction pathway was found of no statistical significance in this analysis, with a *P*-value of 0.47 and an FDR of 0.63.

## Discussion

In the present study, data from both metabolomics and transcriptomics methods were analyzed independently and further integrated. Through these aberrantly-regulated metabolites and genes, we could identify pathways or biological processes altered in the target phenotype, ER stress, thereby unveil the effects it had on lung and provide insights into this cellular state.

PCS, phenol sulphate and 3-oxododecanoic acid accumulated in the lung of ER stress mouse models, yet more metabolites (13, compared to 3 up-regulated) were detected of lower amount than expected, including TMAO. This could indicate the overall elevation of metabolic and anabolic processes inside the body trying to cope with ER stress.

As a uremic toxin, PCS was originally produced by microbes in the gut, the elevation of which in urine has been found associated with worsening outcomes in chronic kidney disease (CKD) patients by fastening the progression of disease as well as increasing cardiovascular burden^25^. In serum samples, PCS has already been identified of significantly lower level in community-acquired pneumonia^26^ and obesity (with unexpected higher level in urine samples)^27^. High oxidative stress caused by exposure to air pollution has been demonstrated by Surya Narayan et al.^28^ to increase urinary PCS in human, this may relate the possible lung damage with PCS elevation detected in this particular study. In an investigation of protozoan-infection-caused acute myocarditis, Núria et al.^29^ have identified PCS as a potential biomarker for the disease by global metabolomic profiling. All in all, though PCS has been found most relative to kidney-related diseases like uremia, kidney failure and CKD (with reference to PubChem), the alarming fold change of 8.45 of this metabolite surely signified prominent changes in the ER-stressed lung, and therefore pose great potential in making PCS a biomarker of ER stress in lung.

Echoing with the lowered level (fold change: 6×) found in the present study, TMAO has long been referred as a chemical chaperone that stabilizes protein conformation against denaturation and even serves to correct folding/trafficking defects of certain protein relative to cystic fibrosis at cellular level^30^, but later proved therapeutically futile as the indices was too narrow in preclinical mouse models^31^. Hu et al.^32^ have identified TMAO as a potential serum biomarker by ^1^H NMR for prediction of non-small cell lung cancer (NSCLC) and evaluating the efficacy of microwave ablation, a preferred treatment of NSCLC. While in a study presented by Klupczynska et al.^33^, TMAO was found to have the highest specificity in discrimination between NSCLC and control samples in the 36 features with statistical significance identified in the serum metabolomics. Similar to PCS, TMAO was proven the downstream product of intestinal microbiota metabolism^34^ and found positively associated with mortality in COPD patients with levels affected also by comorbidities and age^35^. The relation between TMAO and lung diseases has already been noticed and studied. Possibility it had as a biomarker for ER stress is preliminarily demonstrated in our data, but still require further investigation on its specificity comprising other pathologies.

No relative reference regarding lung diseases has presented phenol sulphate (found up-regulated with fold change of 3.62) as disease-signifying marker. In fact, few, if any at all, relation has been made linking this compound with human diseases, possibly due to the large fluctuation in normal-functioning human body as one bioavailability study described its metabolic generation from food polyphenols^36^. Hippuric acid, on the other hand, is very much like TMAO in its dietary origin and relation to microbiota^37^, and its synthesis has been applied clinically as a measurement of liver’s detoxication ability. Its down-regulation (fold change of 5.31) in ER stress phenotype therefore suggested the lowering in hepatic functions and consequently linked liver damage with ER stress.

GSEA results identified phototransduction as the most significantly enriched pathway, including the elevation of genes *Cnga1, Grk1, Calm1* etc. (Supplementary Table S1) in the T group, indicating the pathway’s susceptibility to ER stress. Yu et al.^38^ have also found up-regulated genes enriched in this pathway, in the investigation of lung adenocarcinoma prognosis, revealing the link between phototransduction pathway and pneumopathy. Based on the independent enrichment results of both omics, glutathione metabolism was consistently identified as the pathway of interest. Indeed, Janssen-Heininger et al.^4^ have described glutathione biochemistry prominently associated with ER stress in chronic lung diseases due to the crucial roles of glutathione, as well as its related enzymes and small molecules, play in maintaining the redox homeostasis in cells. It has already been demonstrated by Zhang et al.^39^ that glutathione plays an important role in maintaining the redox homeostasis of ER. In consistence with the existing knowledge, the significance glutathione metabolism exhibits in ER stress has been verified in the present study including the detected elevation of glycine, l-glutamic acid, l-alanine, and oxidized glutathione by the metabolomic methods as well as the 2 up-regulated and 28 down-regulated genes (Supplementary Table S2) with statistically significant variations. Integration of both omics, on the other hand, suggested cancer, infections and multiple signal transduction pathways being influenced by ER stress, demonstrating the close relationship between this particular cellular stress with multiple lung-relating diseases and pointing to future directions in delving this pathogenetic state.

The analysis of PPI revealed much of the influence on the lung by ER stress at molecular and cellular level. For the start, the top 10 nodes selected from the whole PPI network were all members involved in mitochondrial respiratory chain with no exception, and they almost overlap with the top 10 nodes from the top 1 PPI network cluster suggested by MCODE. This indicated both the significance and the great extent to which the cellular respiratory process was altered in ER stress. NADH:ubiquinone oxidoreductase (*Ndufa5*, *Ndufa4, Ndufs6*), ATP synthase (*Atp5j2, Atp5h*) and ubiquinol-cytochrome c reductase (*Uqcr11*) are potential targets identified in these two clusters that could be utilized in detection and remediation of ER stress. The two follow-up PPI network clusters concerned respectively the cell cycle and cancer, as well as G protein coupled receptor (GPCR) and G protein. Like the respiratory pathway pointed out by the first cluster, replication pathways and signal transduction processes are of fundamental importance in maintaining the normal functioning of cells. Cyclin (*Ccnb2*), ubiquitin conjugating enzyme (*Ube2c*) and RAD51 recombinase (*Rad51*) as well as coagulation factor II thrombin receptor (*F2r*), thromboxane A2 receptor (*Tbxa2r*) and tachykinin receptor 1 (*Tacr1*) are few examples of the suggested protein targets with potential in helping against the disease-related condition of ER stress. It should be noted that these targets demand further investigation on their distinguishing ability, sensitivity and availability etc. in the subsequent pursuit of mature therapy or disease screening methods based on ER stress.

The main limitation of the current study is the lack of systematic investigation concerning the selected markers. To further confirm the potential of the markers, more evidence and evaluations should be collected and conducted aiming to reveal the interconnections of these small molecules with the metabolic pathways and specific roles they play in the pathological state of ER stress, but these involve extensive amount of work that is beyond the aim of our study. In the content above, discussion was presented based on the combination of our findings via omic methods and references related to respective small molecules. For the first time, we have shed light on this elusive and disease-associated cellular state of ER stress through methods of metabolomics and transcriptomics, revealing informative molecular alterations as well as presenting potential markers for this cellular state, proposing inspirational interpretations in discussions.

The awareness of prominent roles ER stress plays in lung diseases is growing, yet the complexity and centrality of ER in cells add much difficulty to molecular investigations. By means of metabolomics, we could read the metabolite signature and relate the underlying molecular perturbations to genetic alterations, which was as well obtained through transcriptomics in the present study. Disturbance of glutathione metabolism and the identification of two potential biomarkers, PCS and TMAO, were the two major findings in our study. Integration of metabolome and transcriptome provided reliable insights into the molecular variation of ER stress. The highlighted molecules and pathways here contain great potential yet to be uncovered.

## Methods

### Chemicals and antibodies

Tunicamycin was obtained from Aladdin (T101151-1mg). Phosphate buffered saline (PBS), dimethylsulphoxide (DMSO) and glucose were supplied respectively by Servicebio (G4202-500ML, China), Aladdin (D2650-100ML, China) and SCR/shhushi (63005518, China). The antibodies used in western blot were purchased from companies: anti-β-actin (mouse Ab, 30101ES60, YEASEN, China), anti-CHOP (rabbit Ab, 15204-1-ap, Proteintech, US; CCAAT/enhancer binding protein (C/EBP) homologous protein), and anti-GRP78 (rabbit Ab, 11587-1-ap, Proteintech, US; glucose-regulated protein 78).

### Tissue processing

Eight-week-old male C57BL/6J mice (around 22g) were purchased from Beijing Huafukang Bioscience Co. Ltd. (Beijing, China), grouped [six per group, two groups: C (control) and T (tunicamycin)] and housed in SPF (specific pathogen free) environment (temperature: 22–25 °C, humidity: 60–70%) with 12 h-light/dark cycle and free access to standard rodent pellet food and water. All experimental animals were kept and operated in accordance with the guidelines of the experimental Animal Ethics Committee of the Institute of Materia Medica, Chinese Academy of Medical Sciences. The institutional committee of Animal Ethics in Institute of Materia Medica has approved our experimental protocols. After adaptive feeding for one week, the mice were weighed and injected with tunicamycin (2.5 mg/kg, dissolved in DMSO and diluted with PBS containing 100 mM glucose) or vehicle (PBS with DMSO and glucose) intraperitoneally (i.p.). 12 h after injection, they were anesthetized and sacrificed. Lung tissues were quickly excised, weighed and parted (partially frozen in liquid nitrogen, partially immersed in formalin for pathological sections).

### RNA extraction and real-time PCR

Total RNA extracted using Direct-zol RNA MiniPrep (Zymo Research, U.S.) from 12 lung samples were reversely transcribed to cDNA using cDNA reverse transcription kit (Takara, Japan) and loaded onto quantitative real-time PCR (qPCR, run in triplicates) with TB green (Takara, Japan) and primers (Table 3) synthesized by Beijing AuGCT Biotech Co. Ltd. (Beijing, China). Relative expression levels of genes were quantified using CFX96 real-timesystem (Bio-Rad, Singapore) and normalized in reference to TATA box-binding protein (TBP) expression.

**Table 3:** Primer sequences used in qPCR.

Primer name	Primer sequence (5′–3′)
*mChop*-F	CCACCACACCTGAAAGCAGAA
*mChop*-R	AGGTGAAAGGCAGGGACTCA
*mGrp78*-F	TTCAGCCAATTATCAGCAAACTCT
*mGrp78*-R	TTTTCTGATGTATCCTCTTCACCAGT
*mTbp*-F	ACCCTTCACCAATGACTCCTATG
*mTbp*-R	ATGATGACTGCAGCAAATCGC

F referred to forward primer, while R referred to reverse primer.

### Western blot

Western blot was carried out with lung tissue lysates: sodium dodecyl sulfonate-polyacrylamide gel electrophoresis (SDS-PAGE) and blotting were performed as previously described^40^. Briefly, the lung tissues were lysed in RIPA buffer with protease inhibitor cocktail (CWBIO, China). Equal amount of protein was subjected to SDS-PAGE and immunoblotted with antibodies. Multiple exposures were used to ascertain signal linearity.

### HE staining

Lung tissues that were immersed in formalin were sectioned, stained by hematoxylin and eosin (HE) and photographed using Nikon instruments (ECLIPSE Ti with DIGITAL SIGHT DS-U3).

### Metabolomics analysis

Metabolites were extracted from 6 lung samples each group with accordance to the earlier-described method^41^. ACQUITY UPLC HSS T3 1.8 μm, 2.1 × 100 mm columns (Waters, Dublin, Ireland) were used in the HPLC tandem system of Agilent 1290 II (Agilent Technologies, Germany) and 5600 Triple TOF Plus (AB Sciex, Singapore) as described earlier^42^. Applied mode was electrospray ionization (ESI ±) with condition values listed below: curtain gas = 35, cation mode ion spray voltage = 5500 V, anion mode ion spray voltage = − 4500 V, temperature = 450 °C, ion source gas 1 = 50, ion source gas 2 = 50. Modes of TOF (time of flight) full scan and IDA (information dependent acquisition) were utilized in data collection (impact energy: 30 ± 15 eV). Internal standard molecules include: l-Phenylalanine-D8, l-Tryptophan-D8, l-Isoleucine-D10, l-Asparagine-13C4, l-Methionine-D3, l-Valine-D8, l-Proline-D7, l-Alanine-D7, dl-Serine-D3, dl-Glutamic acid-D5, l-Aspartic acid-D3, l-Arginine-D7, l-Glutamine-D5, l-Lysine-D9, l-Histidine-D5, Taurine-D2, Betaine-D11, Urea-(13C,15N2), l-lactate-13C3, Trimethylamine *N*-oxide-D9, Choline-D13, Malic acid-D3, Citric acid-D4, Succinic acid-d4, Fumaric acid-D4, Hypoxanthine-D3, Xanthine-15N2, Thymidine (13C10,15N2), Inosine-15N4, Cytidine-13C5, Uridine-D2, Methylsuccinic acid-D6, Benzoic acid-D5, Creatine-D3, Creatinine-D3, Glutaric acid-D4, Glycine-D2, Kynurenic acid-D5, l-Citrulline-D4, l-Threonine-(13C4,15N), l-Tyrosine-D7, P-cresol sulfate-D7, Sarcosine-D3, Trans-4-hydroxy-l-proline-D3 and Uric acid-(13C; 15N3). MarkerView 1.3 (AB Sciex, Concord, ON, Canada) was used to retrieve raw data and PeakView 2.2 (AB Sciex, Concord, ON, Canada) was used to align data with standard chemicals and databases (HMDB, METLIN) in recognizing and ascribing metabolites, and analyzing the data in the same time. After evaluating the data using PCA, the up-regulated and down-regulated metabolites were selected based on padj values and fold change (T/C) or fold change reciprocal (C/T, reciprocal of fold change value). The threshold employed was padj < 0.05 and fold change > 2 (or fold change reciprocal < 0.5) and the top 4 metabolites were presented with boxplots. Enrichment analysis, metabolite set enrichment analysis (MSEA), was carried out to view the significant alteration in pathways. The input data were filtered by hypothesis test (Mann–Whitney-U, *P* < 0.05) and rid of metabolites without HMDB ID. The reference database was pathway-associated metabolite sets (SMPDB) and the calibration method was hypergeometric test in over-representation analysis (ORA). The top 20 enriched metabolic pathways were then presented and the significantly enriched pathway was further discussed later in discussion section.

### Transcriptomics analysis

Total RNA was extracted from liquid nitrogen-frozen lung tissues and tested for its integrity using RNA Nano 6000 Assay Kit of the Bioanalyzer 2100 system (Agilent Technologies, CA, USA) before library preparation (via next generation sequencing) for transcriptome sequencing. Library quality was assessed by the Agilent Bioanalyzer 2100 system. RNA-seq was carried out based on Illumina NovaSeq 6000 platform, and sequenced reads were mapped utilizing HISAT2 v2.0.5 while the quantification of gene expression was made by featureCounts (1.5.0-p3). Pearson correlation analysis and PCA were done to evaluate the correlation of samples between groups. Differential expression analysis was carried out in DESeq2 (1.20.0) and volcano plot was made to visualize the differentially expressed genes. The threshold used in identifying aberrantly regulated genes was padj < 0.05 and fold change > 2 (or fold change reciprocal < 0.5). Enrichment analyses of differentially expressed genes were carried out using clusterProfiler (3.4.4). GO, KEGG and Reactome were the 3 databases referred, with different emphasis on gene functions, systematic pathways and biological reactions. Top 30, 20 and 20 pathways enriched were then presented in dot bubble graphs. Conventional enrichment analysis based on hypergeometric distribution relies on significantly up-regulated or down-regulated genes and has risk of leaving out biologically important genes without significant alteration in expression. Gene set enrichment analysis (GSEA), on the other hand, detect changes in the expression of gene sets rather than individual genes, these subtle changes in expression can thus be included. Local GSEA tool (http://www.broadinstitute.org/gsea/index.jsp) was used to do gene set enrichment analysis for the determination of the significance a predefined gene set can present in consistence with the difference between two biological states or phenotypes. Among the pathways from the forementioned 3 databases, the significantly enriched pathway was selected and presented. Based on STRING database, protein–protein interactions (PPI) were analyzed by software Cytoscape. PPI network clusters were established and influential proteins (genes) were identified.

### Statistical analysis, graphics and joint-pathway analysis

All data were expressed as mean ± SEM (standard error of mean). Student's t-test compared the mean values and statistical graphics were constructed by GraphPad Prism 8 software (GraphPad Software Inc., CA, USA). For the selection of differentially expressed genes and metabolites, *P-values* were calculated by t-test and adjusted by calculating padj using the FDR (false discovery rate, BH method) calculation by online platform (http://www.bioinformatics.com.cn/), padj < 0.05 was considered statistically significant. All PCA graphs were made on Orange Data Mining software and volcano graphs on bioinformatics platform (http://www.bioinformatics.com.cn/). Significantly varied metabolites and genes were submitted in running joint-pathway analysis in Metaboanalyst (https://www.metaboanalyst.ca/), relevant graphs were exported from this online tool. Joint-pathway analysis is an integrated metabolic pathway analysis conducted by mapping significant genes together with significant metabolites to metabolic pathways for functional enrichment analysis. By integrating evidence based on changes in both gene expression and metabolite levels, one is more likely to pinpoint the pathways involved in the underlying biological processes, in our case, the ER stress.

### ARRIVE guidelines statement

We confirm that the study was reported in accordance with ARRIVE guidelines (https://arriveguidelines.org).

## Supplementary Information

Supplementary Information.

## Data Availability

The datasets used and analysed during the current study are available from the corresponding author on reasonable request.

## Abbreviations

ER: Endoplasmic reticulum
UPR: Unfolded protein response
COPD: Chronic obstructive pulmonary disease
LC–MS: Liquid chromatography–mass spectrometry
RNA-seq: RNA sequencing
MEK5/ERK5: Mitogen-activated protein kinase 5/extracellular signal-regulated kinase 5
PBS: Phosphate buffered saline
DMSO: Dimethylsulphoxide
Ab: Antibody
CHOP: CCAAT/enhancer binding protein (C/EBP) homologous protein
GRP78: Glucose-regulated protein 78
C/Ctrl: Control (group)
T/Tuni: Tunicamycin (group)
SPF: Specific pathogen free
TBP: TATA box-binding protein
SDS-PAGE: Sodium dodecyl sulfonate-polyacrylamide gel electrophoresis
HE: Hematoxylin and eosin
TOF: Time of flight
ESI: Electrospray ionization
IDA: Information dependent acquisition
GSEA: Gene set enrichment analysis
PPI: Protein–protein interactions
SEM: Standard error of mean
i.p.: Intraperitoneal (injection)
PCA: Principal component analysis
MSEA: Metabolite set enrichment analysis
SMPDB: Database of pathway-associated metabolite sets
GO: Gene ontology
BP: Biological process
CC: Cellular component
MF: Molecular function
KEGG: Kyoto encyclopedia of genes and genomes
NAFLD: Non-alcoholic fatty liver disease
TCA: Tricarboxylic acid
TGF: Transforming growth factor
ZAP: Zeta-chain-associated protein
FDR: False discovery rate
MAPK: Mitogen-activated protein kinase
CKD: Chronic kidney disease
TMAO: Trimethylamine N-oxide
NSCLC: Non-small cell lung cancer
GPCR: G protein coupled receptor
