# Supplementary material for: Untargeted metabolomics and transcriptomics identified glutathione metabolism disturbance and PCS and TMAO as potential biomarkers for ER stress in lung
Source: Sci Rep. 2021 Jul 19;11:14680. doi: 10.1038/s41598-021-92779-8 (PMC8290008; doi:10.1038/s41598-021-92779-8)
Supplement: Supplementary file 1 — Supplementary Information. [file 41598_2021_92779_MOESM1_ESM.pdf]

Supplementary materials:

Figure S1: The cluster selected according to MCODE score of 48.317 (highest), nodes with top 10 MCC scores marked yellow.

Figure S2: The cluster selected according to MCODE score of 27.319 (second highest), nodes with top 10 MCC scores marked yellow.

Figure S3: The cluster selected according to MCODE score of 13.933 (third highest), nodes with top 10 MCC scores marked yellow.

Figure S4: Full length blots from which figure 1 (e) was made: the three pieces (S4 a-c) were from the same gel (adjustments were made to exposure intensity due to different levels of protein expression).

Table S1: Genes probed contributing to the phototransduction pathway.

Table S2: Aberrantly regulated genes enriched in glutathione metabolism.

Table S3. top 20 enriched pathways of MSEA results.

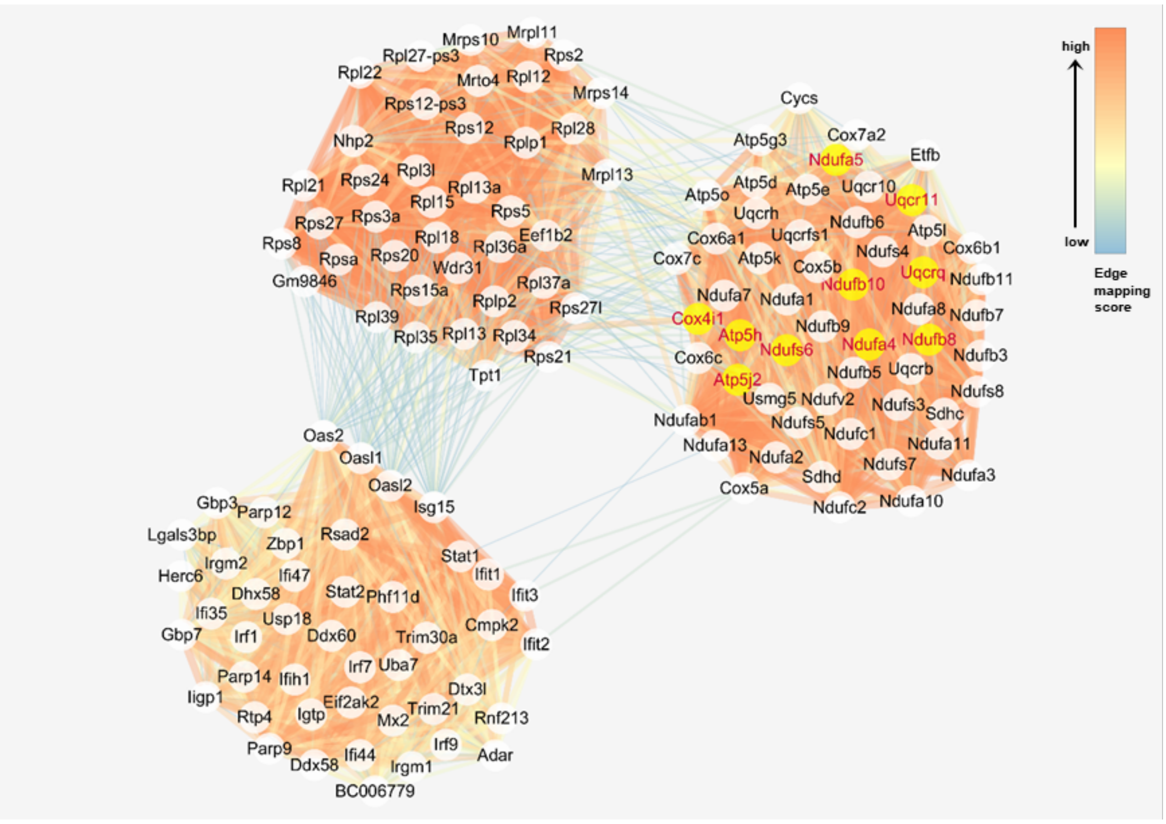

Figure S1. The first cluster selected according to MCODE score of 48.317 (highest), nodes with top 10 MCC scores marked yellow.

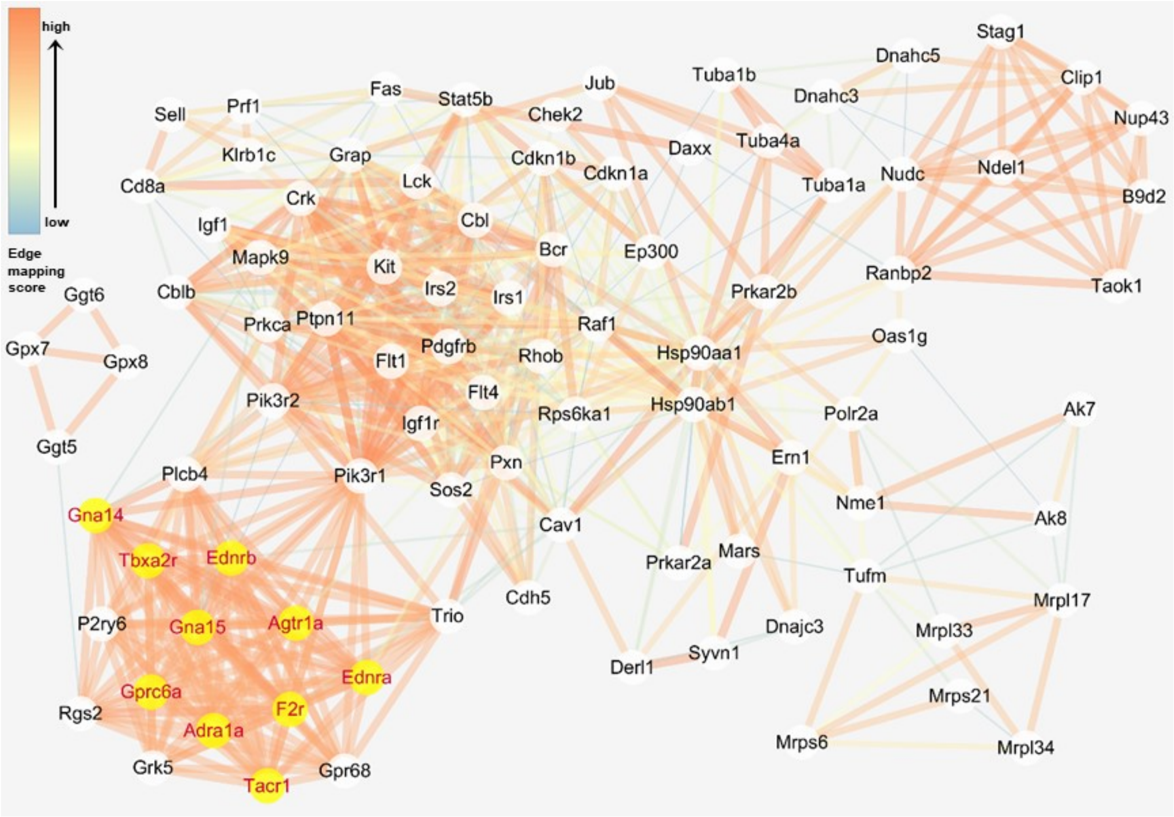

Figure S2. The second cluster selected according to MCODE score of 27.319 (second highest), nodes with top 10 MCC scores marked yellow.

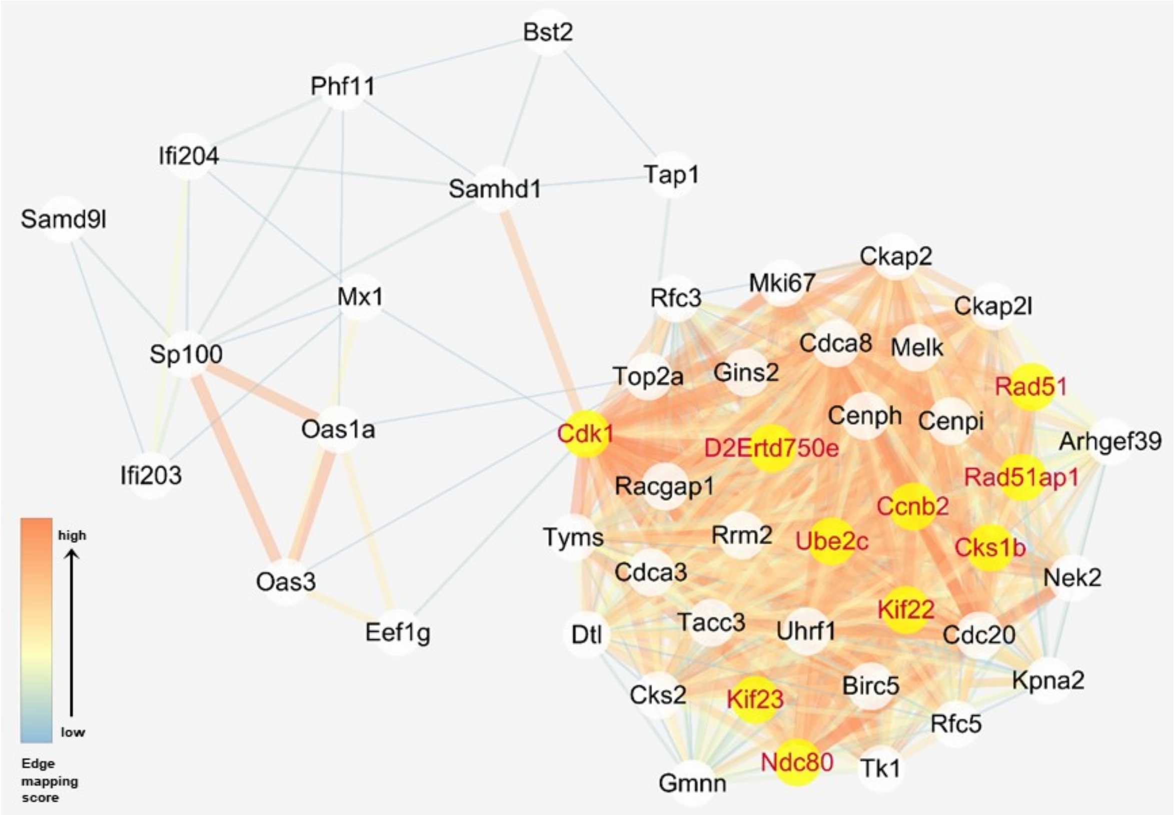

Figure S3. The third cluster selected according to MCODE score of 13.933 (third highest), nodes with top 10 MCC scores marked yellow.

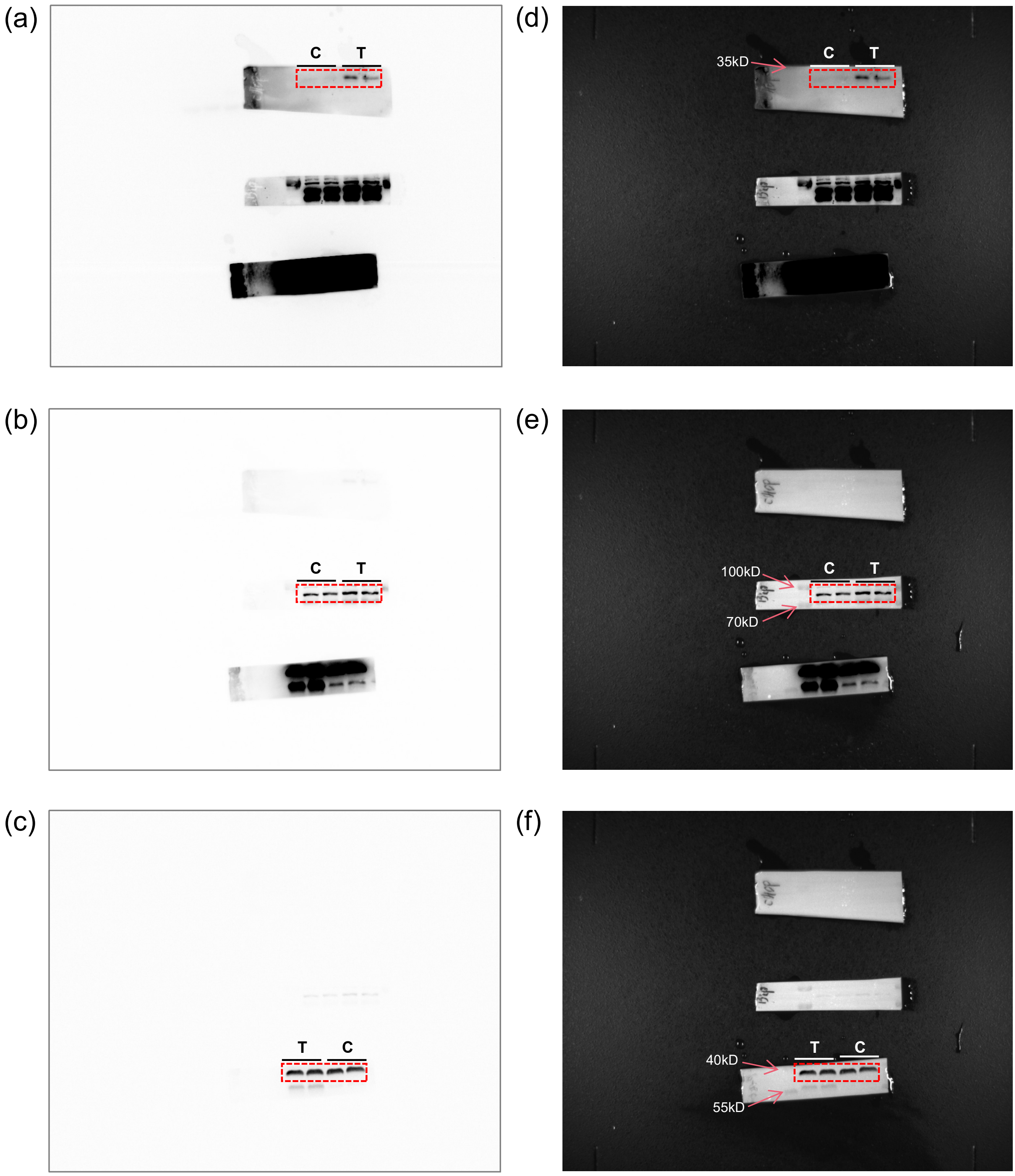

Figure S4. Full length blots from which figure 1 (e) was made (exposure intensity adjusted according to protein amount): CHOP (a, d-merge, expected position: 29kD), GRP78 (b, e-merge, 78kD) and β-actin (c, f-merge, 45kD).

Table S1. Genes probed contributing to the phototransduction pathway.

Gene probe	Running ES	Core enrichment
Cnga1(ENSMUSG00000067220)	-0.6316	Yes
Grk1(ENSMUSG00000031450)	-0.6150	No
Calm1(ENSMUSG00000001175)	-0.6005	Yes
Sag (ENSMUSG00000056055)	-0.5971	No
Gucy2e(ENSMUSG00000020890)	-0.5910	No
Guca1b(ENSMUSG00000023979)	-0.5805	No
Calm4(ENSMUSG00000033765)	-0.5544	Yes
Calm3(ENSMUSG00000019370)	-0.5070	Yes
Guca1a(ENSMUSG00000023982)	-0.4713	Yes
Calm5(ENSMUSG00000099269)	-0.4400	No
Calml4(ENSMUSG00000032246)	-0.3932	Yes
Slc24a1(ENSMUSG00000034452)	-0.2407	Yes
Gucy2d(ENSMUSG00000074003)	-0.2109	No
Gngt1(ENSMUSG00000029663)	-0.1151	No
Pde6b(ENSMUSG00000029491)	-0.0665	No
Gucy2f(ENSMUSG00000042282)	-0.0663	No
Pde6a(ENSMUSG00000024575)	-0.0654	No
Calm2(ENSMUSG00000036438)	-0.0637	No
Rgs9(ENSMUSG00000020599)	-0.0598	No
Rho (ENSMUSG00000030324)	-0.0561	No
Rcvrn (ENSMUSG00000020907)	-0.0510	No
Gnat1(ENSMUSG00000034837)	-0.0478	No
Cngb1(ENSMUSG00000031789)	-0.0473	No
Pde6g(ENSMUSG00000025386)	-0.0292	No
Gnb1(ENSMUSG00000029064)	-0.0261	No
Gnat2(ENSMUSG00000009108)	-0.0225	No
Calml3(ENSMUSG00000063130)	0.0005	Yes

Table S2. Aberrantly regulated genes enriched in glutathione metabolism.

Gene name	Score in ranked gene list	Up / Down regulated
Chac1(ENSMUSG00000027313)	2.25	Up
Gpx3(ENSMUSG00000018339)	1.51	Up
Gsta4(ENSMUSG00000032348)	-1.94	Down
Gsto1(ENSMUSG00000025068)	-1.98	Down
Mgst1(ENSMUSG00000008540)	-1.37	Down
Gstt1(ENSMUSG00000001663)	-1.28	Down
Gpx8(ENSMUSG00000021760)	-1.31	Down
Gstm6(ENSMUSG00000068762)	-1.27	Down
Gpx2(ENSMUSG00000042808)	-1.14	Down
Gpx1(ENSMUSG00000063856)	-0.95	Down
Gss (ENSMUSG00000027610)	-1.22	Down
Gpx4(ENSMUSG00000075706)	-0.92	Down
Gstm5(ENSMUSG00000004032)	-1.29	Down
Mgst2(ENSMUSG00000074604)	-1.22	Down
Idh1(ENSMUSG00000025950)	-0.92	Down
Odc1(ENSMUSG00000011179)	-0.80	Down
Gstp1(ENSMUSG00000060803)	-0.87	Down
Pgd (ENSMUSG00000028961)	-0.85	Down
Rrm2(ENSMUSG00000020649)	-1.28	Down
Ggt5(ENSMUSG00000006344)	-1.05	Down
Ggt6(ENSMUSG00000040471)	-0.93	Down
Anpep (ENSMUSG00000039062)	-0.88	Down
Mgst3(ENSMUSG00000026688)	-0.77	Down
Sms (ENSMUSG00000071708)	-0.86	Down
Gstt3(ENSMUSG00000001665)	-0.80	Down
Gsta2(ENSMUSG00000057933)	-0.78	Down
Gstm2(ENSMUSG00000040562)	-0.74	Down
Gpx7(ENSMUSG00000028597)	-0.83	Down
Gsta1(ENSMUSG00000074183)	-2.68	Down
Gstm4(ENSMUSG00000027890)	-0.96	Down

Table S3. top 20 enriched pathways of MSEA results.

Metabolite Set	total	expected	hits	fold	*P*-value
Glutathione Metabolism	6	1.68	4	2.38	0.0494
Arginine and Proline Metabolism	12	3.35	6	1.79	0.0737
Glutamate Metabolism	10	2.8	5	1.79	0.105
Alanine Metabolism	5	1.4	3	2.14	0.131
Arachidonic Acid Metabolism	5	1.4	3	2.14	0.131
Aspartate Metabolism	8	2.24	4	1.79	0.149
Glycine and Serine Metabolism	14	3.91	6	1.53	0.153
Urea Cycle	11	3.08	5	1.62	0.154
Malate-Aspartate Shuttle	3	0.839	2	2.38	0.188
Phosphatidylcholine Biosynthesis	3	0.839	2	2.38	0.188
Carnitine Synthesis	6	1.68	3	1.79	0.213
Tyrosine Metabolism	6	1.68	3	1.79	0.213
Biotin Metabolism	1	0.28	1	3.57	0.28
Folate Metabolism	1	0.28	1	3.57	0.28
Inositol Metabolism	1	0.28	1	3.57	0.28
Porphyrin Metabolism	1	0.28	1	3.57	0.28
Starch and Sucrose Metabolism	1	0.28	1	3.57	0.28
Vitamin B6 Metabolism	1	0.28	1	3.57	0.28
Methionine Metabolism	10	2.8	4	1.43	0.289
Warburg Effect	10	2.8	4	1.43	0.289
